# Scaffolding during the cell cycle by A-kinase anchoring proteins

**DOI:** 10.1007/s00424-015-1718-0

**Published:** 2015-07-24

**Authors:** B. Han, W. J. Poppinga, M. Schmidt

**Affiliations:** Department of Molecular Pharmacology, University of Groningen, Groningen, The Netherlands; Groningen Research Institute for Asthma and COPD, GRIAC, Groningen, The Netherlands

**Keywords:** AKAPs, Scaffolding, Cell cycle, Proliferation, Cancer

## Abstract

Cell division relies on coordinated regulation of the cell cycle. A process including a well-defined series of strictly regulated molecular mechanisms involving cyclin-dependent kinases, retinoblastoma protein, and polo-like kinases. Dysfunctions in cell cycle regulation are associated with disease such as cancer, diabetes, and neurodegeneration. Compartmentalization of cellular signaling is a common strategy used to ensure the accuracy and efficiency of cellular responses. Compartmentalization of intracellular signaling is maintained by scaffolding proteins, such as A-kinase anchoring proteins (AKAPs). AKAPs are characterized by their ability to anchor the regulatory subunits of protein kinase A (PKA), and thereby achieve guidance to different cellular locations via various targeting domains. Next to PKA, AKAPs also associate with several other signaling elements including receptors, ion channels, protein kinases, phosphatases, small GTPases, and phosphodiesterases. Taking the amount of possible AKAP signaling complexes and their diverse localization into account, it is rational to believe that such AKAP-based complexes regulate several critical cellular events of the cell cycle. In fact, several AKAPs are assigned as tumor suppressors due to their vital roles in cell cycle regulation. Here, we first briefly discuss the most important players of cell cycle progression. After that, we will review our recent knowledge of AKAPs linked to the regulation and progression of the cell cycle, with special focus on AKAP12, AKAP8, and Ezrin. At last, we will discuss this specific AKAP subset in relation to diseases with focus on a diverse subset of cancer.

## Introduction

The growth of organisms is driven by cell division which relies on coordinated regulation of phases in cell cycle [4]. When the cell is quiescent, it remains in the G1 phase; however, on initiation of cell division, it progresses into the S phase, during which DNA replication occurs, followed by a separation of sister chromatids during the M phase, which in turn is again separated in the pro-, meta-, ana-, and telophase, followed by cytokinesis where the actual cell division occurs. A series of strictly regulated molecular mechanisms are required to maintain the progression of the cell cycle including cyclin-dependent kinases (CDKs), retinoblastoma protein (Rb), and polo-like kinases (Plks) [10, 24, 117]. In accordance, several diseases have been found to directly or indirectly relate to a defective regulation of the cell cycle, such as cancer, diabetes, and neurodegeneration [93, 115].

Importantly, over the past years, it is accepted that compartmentalization of cellular signaling is a common strategy used to ensure the accuracy and efficiency of cellular responses [67]. Cellular compartmentalization of proteins involved in signal transduction is maintained by scaffolding proteins, such as A-kinase anchoring proteins (AKAPs), which are able to orientate a diverse subset of signaling effectors, such as protein kinase A (PKA), extracellular-signal-regulated kinase (ERK), and cyclins, towards selected substrates in specific cellular microdomains [80, 91, 109]. AKAPs are characterized by their ability to anchor the regulatory subunits of PKA via a conserved short α helical structure, and thereby achieve guidance to different cellular locations via various targeting domains. PKA is a cAMP-dependent serine/threonine kinase and a very important player in many different cellular pathways. The involvement of PKA in the cell cycle progression is diversely regulated in different cell types. In *Xenopus* embryonic cells, PKA activity is low during the M phase but increases during M/G1 transition, [36, 37], whereas in the human cancer cell line HeLa, PKA activity is increased during the M phase [104]. PKA negatively regulates the cell cycle progression upon activation of the small GTPase Rap1 and subsequent sequestration of Ras/MEK/ERk [22, 89]. Inhibition of the cell cycle progression by PKA can also be achieved upon upregulation of the CDK inhibitor p27Kip1 [46].

Next to PKA, AKAPs also associate with several other signaling elements including receptors, ion channels, protein kinases, phosphatases, small GTPases, and phosphodiesterases [23, 80, 91]. Until now, over 50 members of the AKAP family have been identified, and each AKAP can form a unique signaling complex in different microdomains in the cells [29, 80, 91, 103]. With the large variety of AKAP signaling complexes at many different locations inside the cell, it is feasible that such AKAP-based complexes regulate several critical cellular events of the cell cycle. In fact, several AKAPs are assigned as tumor suppressors due to their vital roles in cell cycle regulation.

Although the function of AKAP-PKA interactions in the cell cycle is not well understood, the role of some AKAPs being unveiled and will be described in this review. Here, we first briefly discuss the most important players of cell cycle progression. After that, we will review our recent knowledge of AKAPs linked to the regulation and progression of the cell cycle, with special focus on AKAP12, AKAP8, and Ezrin. In the final section, we will discuss more about AKAP12 and Ezrin in relation to disease.

## Players of cell cycle regulation

**Fig. 1:**
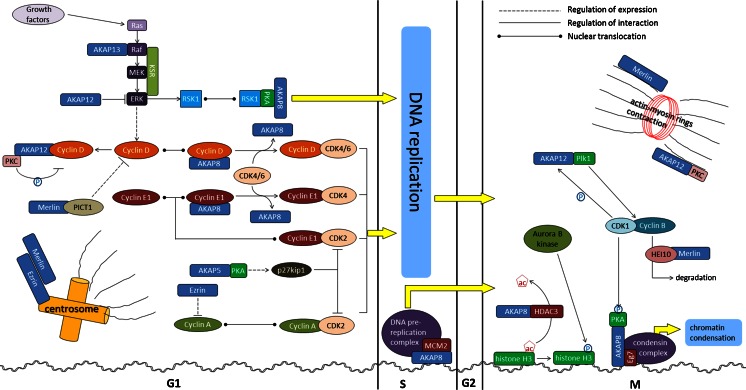
A-kinase anchoring proteins regulate the cell cycle by spatial and temporal interaction with several key players. With the initiation of the G1 phase, cyclin-CDK signaling is crucially mediated by several AKAPs, most notably AKAP5, AKAP8, and AKAP12. AKAPs can mediate this by controlling either the expression, nuclear translocation, or activity of the cyclins and/or CDKs. During the S phase, only the interaction of AKAP8 with the DNA replication complex is known. Throughout the different stages of the M phase AKAPs, again mainly AKAP8 and AKAP12, play a role in coordinating the initiation and finalizing of different stages from chromatin condensation until the cytokinesis that is at the very end. AKAP12 regulates the cell cycle by (**a**) acting as a negative regulator during inappropriate cell cycle progression and n**b**) supporting mitosis and cytokinesis. As illustrated, AKAP12 decreases cyclin D expression via ERK. AKAP12 binds also to cyclin D1 to prevent its nuclear translocation. On the other hand, AKAP12 supports cytokinesis completion by controlling actin-myosin rings via scaffolding of PKC and actin. In addition, AKAP12 forms a complex with the mitotic kinase Plk1, known to activate the cyclin B-CDK1 complex during G2/M phase transition. As the only identified AKAP within nucleus, AKAP8 functions as a multivalent platform to anchoring different signaling elements during cell cycle regulation. AKAP8 helps to recruit MCM2 to DNA and promotes thereby its replication during S phase. In the presence of growth factors, AKAP8 localizes to ERK-induced RSK1 in the nucleus to subsequently induce cell proliferation. Moreover, AKAP8 supports the delivery of cyclin D/E to CDKs and thereby facilitates cell cycle progression. During the M phase, AKAP8 recruits HDAC3 to the vicinity of chromatin and thereby initiates chromatin condensation. Subsequently, AKAP8 localizes the condensin complex to chromatin and initiates thereby chromatin condensation. Together with Merlin, Ezrin helps centrosome positioning and thereby guides mitotic spindle orientation during cell division. AKAP8 also decreases cyclin A expression by acting as a transcriptional repressor, and thereby reduces cell proliferation. AKAP5 modulates cell proliferation by affecting the expression of a specific CDK2 inhibitor p27kip1. Together with KSR-1, AKAP13 forms a scaffolding core, thereby allowing tuning of ERK signaling. Merlin controls cyclin B1 levels by HEI10 localization. Merlin seems also to repress cyclin D1 expression through its interaction with another tumor suppressor, PICT-1. For further details, abbreviations and references, see text

The cell cycle is controlled by the activity of CDKs, which in turn are controlled by cyclins such as cyclin D/E [112]. Exposing cells to growth factors will elevate the amount of cyclins e.g., cyclin D1 in the cell through the Ras/Raf/MEK/ERK signaling cascade, [16, 76, 82], which can combine with pre-existing CDKs to activate or inactivate target proteins, such as Rb, to orchestrate the entry into the different phases of the cell cycle [74]. The activity of cyclin-CDK complexes is tightly controlled, as check points, to fine-tune the cell cycle. For example, Plk1 activates cyclin B-CDK1 complex, during the prophase to initiate the G2/M transition [100, 101]. In addition, also the degradation of cyclins by ubiquitination allows cells to enter a next phase of the cell cycle. For example, human enhancer of invasion 10 (HEI10) functions as an E3 ubiquitin ligase to inhibit the progression into the M phase by decreasing the levels of cyclin B [99]. In addition, the M phase is regulated by a series of complexes or enzymes that control chromosome segregation and condensation (e.g., condensin, histone H3, and Aurora B kinase) [42, 58, 108]. In Fig. 1, the interactions between AKAPs and several key players in cell cycle regulation are summarized.

## AKAP12

AKAP12, originally called Gravin or AKAP250, was first recognized as an autoantigen in serum from myasthenia gravis patients [35]. Later, AKAP12 was found orthologous to a rodent protein, the Src-suppressed C Kinase Substrate (SSeCKS) [62]. Since its discovery as an AKAP [73], AKAP12 is probably one of the most studied AKAPs in the cell cycle regulation [34]. Several reports indicate that AKAP12 generally regulates the cell cycle upon engagement of distinct cell cycle phases: (1) acting as a negative regulator during inappropriate cell cycle progression; (2) helping to facilitate mitosis and cytokinesis [1, 73].

### AKAP12 as a negative regulator of the G1/S transition

AKAP12 regulates the cell cycle by reducing cyclin D1 expression presumably mediated via inhibition of ERK [16, 82, 97, 105]. For example, Lin et al. reported in NIH 3T3 cells that induction of AKAP12 expression by tetracycline suppressed ERK2-dependent cyclin D1 expression and Rb phosphorylation, effects that coincided with a G1 arrest [60]. Conversely, knockdown of AKAP12 in a C6 rat glioma cells reversed dexamethasone-induced growth arrest, which was associated with elevated phosphorylation of ERK1/2 and expression of cyclin D1 [63]. Regulation of ERK might be mediated by controlling Src-focal adhesion kinase (FAK) complexes. AKAP12 sequesters Src through direct binding [61, 95], thereby disengaging Src-FAK complexes away from ERK [1, 34]. Besides affecting the expression of cyclin D1, AKAP12 also sequesters cyclin D1, and thereby inhibits its nuclear translocation. In AKAP12-overexpressing NIH 3T3 cells, the majority of cyclin D1 co-localized with AKAP12 in the cytoplasm, and nuclear cyclin D1 was reduced by about 70 % compared to controls [60]. In agreement, Burnworth et al. showed that cell-cell contact-induced AKAP12 expression sequesters cyclin D1 and prevents its nuclear translocation, resulting in growth arrest of glomerular parietal epithelial cells [12]. AKAP12-mediated cyclin D1 sequestration is inhibited by protein kinase C (PKC) phosphorylation of AKAP12 [59] and short-term activation of PKC induced nuclear translocation of cyclin D1 even under AKAP12-overexpression (Fig. 1) [60].

### AKAP12 is important for the completion of cytokinesis

AKAP12 regulates cell cycle progression by facilitating the cytokinesis. In HeLa cells, Choi et al. found that knockdown of AKAP12 resulted in a profound rounding up of the cell morphology and multi-nucleated cells at later stages of cytokinesis compared to controls, leaving the anaphase and telophase of the cell cycle unchanged [19]. Similarly, AKAP12-deficient mouse embryonic fibroblasts proliferated faster compared to wild-type cells in early passages, but lost all proliferative capacity during later passages and showed significant Rb-dependent cell senescence and multi-nucleation [2]. AKAP12 is known to bind to PKC, thereby decreasing PKCα and δ activity [2, 40]. PKCα can lead to cell senescence by activation of p16INK4a/Rb [96], while PKCδ causes the downregulation of Lats1/Warts, a mitotic exit network kinase required for completion of cytokinesis [45, 111]. In addition, in HEK293 cells, AKAP12 was found to co-localize with actin near the actin-myosin contractile rings known to be important to complete cytokinesis (Fig. 1) [19]. Reports have shown that PKC completes cytokinesis upon contraction of the actin-myosin ring [9, 87]. AKAP12 might be involved in the regulation of cytokinesis by controlling the contraction of actin-myosin rings by scaffolding PKC and actin.

Recently, AKAP12 was also found to form a complex with a mitotic kinase Plk1 (Fig. 1) [13], known to activate the cyclin B-CDK1 complex during G2/M phase transition [100, 101]. Interestingly, phosphorylation of AKAP12 by CDK1 was required for the binding of Plk1 to AKAP12 (Fig. 1). Taking together, AKAP12 may amplify the formation of cyclin B-CDK1 complexes due to its ability to function as a scaffolding protein for Plk1. As support, disruption of AKAP12-Plk1 using a Plk1-binding deficient AKAP12 mutant decreased cell proliferation [14]. Conclusively, AKAP12 may play an important role in cell cycle regulation and may thus represent a potential target for the treatment of cancer and other proliferation-associated diseases.

## AKAP8

AKAP8, also known as AKAP95, is an AKAP that has been identified to reside in the nucleus, which leaves no surprise that AKAP8 is involved in DNA replication and the expression levels of several proteins that regulate the cell cycle (Fig. 1) [91].

### AKAP8 in interphase

As outlined below, various studies suggest AKAP8 regulates the cell cycle through its interaction with different proteins during interphase, such as minichromosome maintenance protein 2 (MCM2), ribosomal S6 kinase 1 (RSK1), and cyclin D/E (Fig. 1). Using a yeast two-hybrid screening, one of the AKAP8-binding proteins identified in HeLa cells was MCM2, a component of the DNA pre-replication complex, which is in charge of DNA replication [28, 56]. Disruption of the AKAP8-MCM2 interaction by GST-AKAP8 peptides decreased or even abolished DNA replication. In S phase nuclei, depletion of chromatin-associated AKAP8 by partially removed MCM2 and inhibited the initiation and elongation phases of DNA replication [28], suggesting that AKAP8 plays a central role in controlling MCM2 function.

Activation of RSK1 by ERK1/2 leads to its subsequent translocation to the nucleus, phosphorylation of downstream substrates, and growth factor-induced proliferation [6]. In HeLa cells, Gao et al. discovered that an AKAP-PKA interaction inhibitor, stearated Ht31, reduced epidermal growth factor-induced RSK1 nuclear translocation [32]. Taken together, these data indicate that AKAPs are involved in the nuclear retention of RSK1. Combining immuno-precipitation and liquid chromatography-mass spectrometer analyses, AKAP8 was indeed identified as the AKAP responsible for this nuclear retention of RSK1. Supportively, silencing of AKAP8 decreased nuclear RSK1 and increased cytosolic RSK1 (Fig. 1) [32].

In Chinese hamster ovary cells, AKAP8 could co-immunoprecipitate with cyclin D [8] and cyclin E1 [7] (Fig. 1). Interestingly, cyclin D/E were found to combine with either AKAP8 or CDK4, indicating a competition for cyclin binding [7]. Interactions between AKAP8 and cyclin D/E were impaired upon overexpression of CDK4 in the cells [7, 8], suggesting that AKAP8 may help to deliver cyclin D/E to CDK4 to facilitate cell cycle progression. As cyclins require to be combined with a distinct subset of CDKs to exert a regulatory function on the cell cycle [112], these findings provide another mechanism for AKAP8 to regulate the cell cycle.

### AKAP8 is important for chromatin condensation

It is reported that AKAP8 helps to regulate chromatin condensation by interacting with the DNA and other proteins during the mitotic phase. In HeLa cells, Steen et al. found, by Western blot analysis of the nuclear matrix and chromatin fractions prepared at different phases of the cell cycle, that AKAP8 redistributed from the nuclear matrix to the chromatin upon mitotic nuclear disassembly [94]. Meanwhile, AKAP8 was found to directly interact with a human condensin complex component, Eg7, and thereby to support its recruitment to chromatin (Fig. 1) [21, 94]. The data suggest that AKAP8 regulates the M phase by localizing the condensin complex to chromatin via its direct interaction with Eg7 during chromatin condensation.

Chromatin condensation is initiated by the phosphorylation of histone H3 serine 10 by Aurora B kinase [42, 108]. This phosphorylation is regulated by AKAP8 during chromatin condensation. When cells enter the M phase, AKAP8 was found to recruit histone deacetylase 3 (HDAC3) to the vicinity of chromatin. The de-acetylation of histone H3 by HDAC3 resulted in a hypo-acetylated tail, which became a preferred substrate for Aurora B kinase, allowing phosphorylation of histone H3 at serine 10 [58]. In agreement, depletion of either AKAP8 or HDAC3 induced G2/M arrest and substantially increased cells with incomplete chromosomal condensation, defects in chromosome segregation, and tri- or multipolar mitotic spindles [58].

Although PKA has been implicated as a negative regulator for proliferation in several cell types such as airway smooth muscle cells, vascular smooth muscle cells, NIH 3T3 cells, and adipocytes [11, 15, 44, 88], other studies found that PKA activity relatively increased during the M phase [36, 90]. Collas et al. found that Ht31, anti-AKAP8 antibodies, the PKA inhibitors PKI or Rp-8-Br-cAMPS induced premature chromosome de-condensation [21], suggesting that AKAP8-anchored PKA activity is essentially required for condensed chromatin maintenance during the M phase. Further studies indicated that PKA-AKAP8 anchoring requires phosphorylation of PKA regulatory subunit IIα at threonine 54, as a PKA regulatory subunit IIα T54E mutant impaired in phosphorylation, inhibited interaction between PKA and chromatin-associated AKAP8 during the M phase [54]. The cyclin B-CDK1 complex seems to be involved in this process, as it had been found to phosphorylate at threonine 54 of PKA RIIα during the M phase (Fig. 1) [50].

## Ezrin

Identified as an AKAP [25], Ezrin is a member of the Ezrin, Radixin, and Moesin protein family, this family crosslinks the membrane with its underlying actin cytoskeleton and helps to regulate a diverse subset of signaling routes [30]. Although many studies have related Ezrin with cancer metastasis and invasion [53, 64, 69, 83], data also suggested that Ezrin may play a role in cancer by regulating the cell cycle (Fig. 1) [18, 41, 51, 85, 86].

Ezrin was found to direct mitotic spindle orientation during cell division [41]. Hebert et al. showed that Ezrin concentrated at certain areas of the plasma membrane to form a cap-like structure to help centrosome positioning, starting during G1 and reaching a peak by the S phase [41]. Interestingly, Ezrin acts in concert with the closely related neurofibromatosis type II (NF2) tumor suppressor Merlin to exert this function [41], which was also identified as an AKAP (see below) (Fig. 1) [39]. In cells expressing Merlin short hairpin RNA, cortical Ezrin fails to form a cap-like structure, resulting in aberrantly oriented spindles and polarization [41].

Besides functioning as a cytoskeletal protein and cortical cue to direct mitotic spindle orientation, Ezrin seems to participate also in cell cycle regulation by acting as a transcriptional repressor. In endothelial cells, TNF-α induced a downregulation of cyclin A and decreased cell proliferation, which seemed to be mediated by the nuclear recruitment of an 84-kDa protein, that bound specifically to the cell cycle genes homology region in the cyclin A promoter [52], which was later identified as Ezrin [51]. Conversely, endothelial cells transfected with dominant-negative Ezrin largely attenuated TNF-α-induced downregulation of cyclin A promoter activity and inhibition of proliferation. In a mouse hind limb ischemia model, transplantation of dominant-negative Ezrin-transfected endothelial cells improved blood flow recovery by increased endothelial cell proliferation [51]. It is known that cytoskeletal organization of Ezrin involves the Rho family of GTPases [110]. Interestingly, the same study also found that the TNF-α-induced Ezrin expression needed activation of RhoA kinase [51]. Supportively, similar effects of Ezrin were also discovered in another system, where fibroblast growth factor induced Ezrin expression resulted in growth arrest in the G1 phase in rat chondrosarcoma cells [85].

In cancer cells however, Ezrin seems to act differently as it plays a critical role during tumor progression by positively regulating the cell cycle progression. In tongue squamous cell carcinoma (SCC), high Ezrin expression correlated with an increased Ki-67 index, a marker for tumor proliferation and aggressiveness, although no obvious connection between the expression level of Ezrin and the tumor stage was observed in this study [86]. Furthermore, Ezrin was found to be involved in cancer proliferation by affecting cell cycle distribution, as silencing of Ezrin decreased the S and G2/M fractions and the growth rate in human tongue SCC cell line HSC-3 [86]. Similarly, in human lung cancer cell line 95D, Ezrin short hairpin RNA arrested the cells in G0/G1 phases, which lead to the delay of cell cycle progression and inhibited cell proliferation [18].

## Other AKAPs

### AKAP5

AKAP5, also known as AKAP79, has been reported to reduce cell proliferation by increasing the expression of p27kip1, a specific CDK2 inhibitor, in a PKA dependent way (Fig. 1) [46]. In line with this function, AKAP has been found in the nuclear fraction [114]. In rat aortic smooth muscle cells, Indolfi et al. found that overexpression of AKAP5 resulted in high cAMP-dependent signaling, a process most likely relying on the association of membrane-bound AKAP5 with PKA, as the cAMP signaling was diminished by co-expression of the PKA inhibitor PKI or a derivative of AKAP5 without the membrane-anchoring domain. Enhanced transcriptional activity of the cAMP-dependent CRE promotor by AKAP5 was correlated with a high p27kip1 expression and low DNA synthesis level [46]. Supportively, in a rat vascular injury model, site-specific gene transfection of AKAP5 after balloon injury significantly increased the p27kip1 level and inhibited neo-intimal hyperplasia [46].

### AKAP13

Through the Raf/MEK/ERK cascade, the ERK pathway transduces signals from growth factor-stimulated membrane receptors to growth factor-responsive targets in the cytosol and nucleus [81, 106]. It is already known that kinase suppressor of Ras (KSR) acts as a scaffolding protein to modulate the ERK signaling network (Fig. 1) [98]. Another study indicated that besides KSR, AKAP13, also known as AKAP-Lbc, is also involved in this signaling complex (Fig. 1) [92]. Using HEK293 cells and NIH 3T3 fibroblasts, Smith et al. elucidated a molecular model, in which AKAP13 and KSR form a scaffolding core to localize Raf in the vicinity of MEK, allowing a signaling cascade from Raf, through MEK, to ERK1/2 [92]. The growth factor induced Raf/MEK/ERK cascade happens during the G1/S transition [27, 68], pointing towards AKAP13 playing a role during this transition. More importantly, this molecular model seems to suggest a reasonable explanation for the positive effects of PKA on the ERK cascade and cell proliferation [26], as the function of this signaling complex was depended on the phosphorylation of Serine 838 on KSR by an AKAP13 anchored PKA (Fig. 1) [92].

### Merlin

As mentioned previously, Merlin is an AKAP that anchors different signaling proteins to the actin cytoskeleton and involves in cell signaling during cell proliferation [39]. Because the mutation of human Merlin gene is known to cause NF2, Merlin is also called neurofibromin 2 or schwannomin [84, 102]. As with Ezrin, it was found that cellular localization of Merlin was dependent on the cell cycle [72]. Merlin was found accumulated around the nucleus at the G2/M transition, but localized to mitotic spindles and the contractile ring during the M phase, and later more, Merlin was found underneath the cortical membrane during the G1/S phases [72]. PKA phosphorylation coordinates a lot of Merlin’s functions as PKA phosphorylation of Merlin at serine 10 is required for its interaction with the actin cytoskeleton [55]. In addition, PKA phosphorylation of Merlin at serine 518 causes a heterodimerization with Ezrin [5], directing mitotic spindle orientation during cell division [41]. The growth inhibition effects of Merlin are linked to its regulation of cyclin B or D1 (Fig. 1). In a rat schwannoma cell line, Grönholm et al. found that Merlin expression is necessary for the subcellular localization of HEI10 [38], a protein controlling the levels of cyclin B1 by acting as a divergent class of E3 ubiquitin ligase (Fig. 1) [99]. In addition, Merlin was suggested to repress cyclin D1 expression through its interaction with another tumor suppressor, protein interacting with carboxyl terminus 1 (PICT-1) in glioblastoma cells [17].

## Relation to diseases

In the next section, we highlight some aspects of AKAP12, Ezrin, and Merlin in a disease-related context. We summarized the involvement of AKAPs in various diseases in Table 1. AKAP12 gene is mapped to 6q24-25.2, which is a hotspot for gene deletions during cancer progression [33, 34]. Downregulation of AKAP12 expression has been reported to cause abnormal cell cycle regulation, leading to pulmonary adenocarcinoma [107], prostatic hyperplasia [3], myelodysplastic syndrome [77], and gastric carcinoma [20]. Data indicate that gene silencing of the AKAP12 promoter through CpG island hypermethylation is responsible for the downregulation of AKAP12 in esophageal neoplastic progression [48], colon cancer [70], and gastric carcinoma [20], suggesting that hypermethylation of the AKAP12 promoter may represent a potential indication for the early detection of a distinct subset of diseases. On the other hand, restoration of AKAP12 expression might be beneficial in future treatment of cancer. Indeed, re-expression of AKAP12 in gastric cancer cells restored cell growth by inducing apoptosis [20]. Similarly, re-expression of AKAP12 suppressed the ability of v-Src to induce cell growth and induced cell arrest in an AKAP12 deficient cell line [61].

**Table 1:** AKAPs and diseases

AKAPs	Diseases	References
AKAP12	Pulmonary adenocarcinoma	[89]
	Prostatic hyperplasia	[3]
	Myelodysplastic syndrome	[64]
	Esophageal neoplastic progression	[38]
	Colorectal cancer	[58]
	Gastric carcinoma	[19]
Ezrin	Uterine cervical cancer	[43]
	Uveal malignant melanoma	[54]
	Tongue squamous cell carcinoma	[72]
	Hepatocellular carcinoma	[39]
	Brain astrocytoma	[55]
	Atypical endometrial hyperplasia	[62]
	Uterine endometrioid adenocarcinoma	[63]
	Colorectal cancer	[46]
	Lung cancer	[17]
Merlin	Neurofibromatosis type II	[70, 85]
	Melanoma	[59]
	Mammary tumor	[35]
	Osteosarcoma	[35]

For further details, see text

Unlike AKAP12, Ezrin expression and increased malignancy seem to correlate in various human cancers, including uterine cervical cancer [53], uveal malignant melanoma [65], tongue SCC [86], hepatocellular carcinoma [49], brain astrocytoma [66], and atypical endometrial hyperplasia and uterine endometrioid adenocarcinoma [75]. This suggests that Ezrin expression could be a potential prognostic marker for these diseases. In accordance, Ezrin knockdown by silencing RNA decreased cell proliferation and survival rate in tongue SCC cell line [86], human lung cancer cell lines [18], and colorectal cancer cell lines [57]. Moreover, inhibition of Ezrin expression seem to reduce the chemotherapy resistance of human lung cancer cells [18], suggesting a potential AKAP-related strategy for this disease. Notably, Ezrin phosphorylation is necessary for cancer cell proliferation. There is an increased Ezrin phosphorylation at threonine 567 in liver metastasis compared to the primary tumor. Interestingly, overexpression of T567D Ezrin, a phospho-mimicking Ezrin mutant, promoted the cancer cell proliferation [57, 116], while an overexpression of wild-type Ezrin showed inhibitory effects on cell proliferation [116].

The most studied disease that relates to Merlin is NF2, as it is caused by mutations of the Merlin gene [84, 102]. Later studies suggested that the mechanism behind its tumor suppressor properties may also applies to other type of cancers [71]. The tumor suppression mechanism of Merlin is mainly associated with contact-mediated growth inhibition. At high cell density, Merlin was found hypo-phosphorylated and its growth-inhibitory activity was depended on interaction with the cytoplasmic tail of CD44 [71]. In addition, Merlin is also suggested to prevent centrosome amplification during tumorigenesis, as loss of Merlin fails to restrict Ezrin, leading to incorrect centrosome position and multipolar spindle formation in Merlin-deficient Caco2 cells, BT-549 mammary tumor cells and U2OS osteosarcoma cells [41]. For a comprehensive understanding of the role of Merlin in tumors, the authors recommend the latest review [78].

## Conclusion

In conclusion, there are several indications that AKAPs can regulate the cell cycle through either participating in signaling pathway by themselves or functioning as the scaffolding proteins that anchor and coordinate different signaling elements (Fig. 1).

**Fig. 2:**
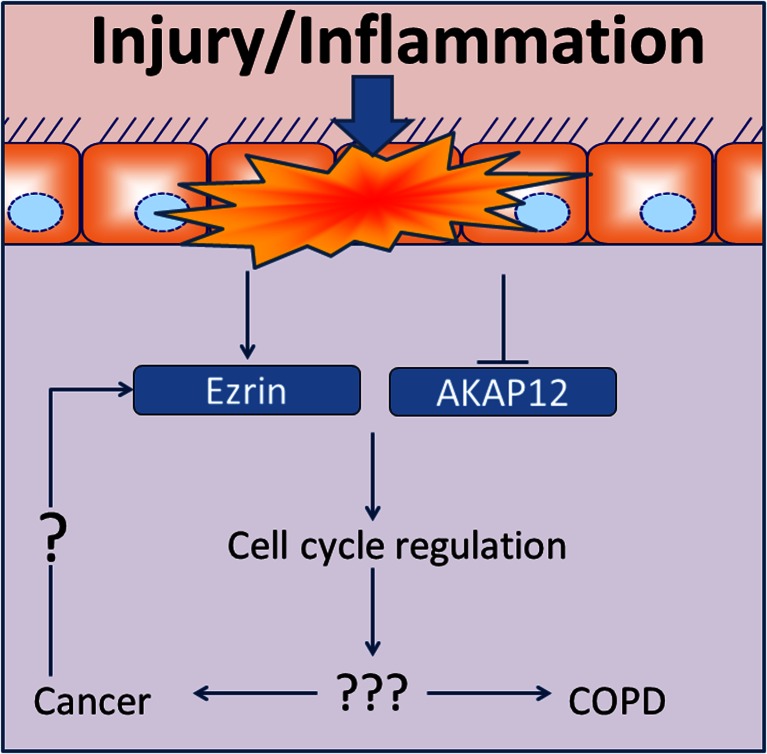
Balance between a distinct subset of AKAPs important for disease development. Expression of AKAP12 is decreased in various cancers and COPD. In cancers, Ezrin switched from an anti-proliferative to a pro-proliferative function. Cigarette smoke induced reduction of AKAP12 and elevation of Ezrin in airway smooth muscle might represent a common link between cancer and CODP. For further details and references, see text

This review has focused only on a few AKAPs, which have been shown to link cell cycle alterations and disease (Table 1). It is tempting to speculate that their balance could play an important role in other diseases through a yet to be defined mechanism. In this context, it is worthwhile to mention that we have shown recently that cigarette smoke, a major cause not only for lung cancer but also for chronic obstructive pulmonary disease (COPD), provoked a decrease of AKAP12 and an increase of Ezrin expression in airway smooth muscle [79]. Although both AKAP12 and Ezrin seem to inhibit proliferation, as outlined in detail above Ezrin, seems to change its function in cancer. Thus, it would be interesting to study if Ezrin alters its function also in COPD. Taken together with what we have discussed above, it seems that understanding the balance of these AKAPs could be important to unravel basic mechanisms underlying a variety of diseases (Fig. 2).

Several issues regarding to AKAPs in cell cycle regulation need to draw more attention. First, all AKAPs can bind to PKA, but the role of PKA in AKAP-mediated cell cycle regulation is still unclear. Second, some AKAPs were reported to interact with the same signaling partners (e.g., cyclin B), what could the mechanism be to coordinate different AKAPs to interact with the same signaling partner? In the case of PKA, AKAPs have varying affinities for the enzyme, which can be affected by the activation of PKA [43, 113]. For example, Ezrin binds PKA (RII) only with low affinity [47]. Therefore, it is tempting to speculate that an AKAP with a higher affinity for PKA can compete with PKA binding to an AKAP with a lower affinity for PKA. However, this is thus far unstudied. There have been limited studies addressing the options of AKAPs affecting each other, however, it has been published that AKAP5 and AKAP12 can form heterodimers [31]. The authors showed that overexpression of AKAP12 in cells that endogenously express AKAP5, such as HEK293 or A431 cells, potentiates AKAP5-mediated phosphorylation of ERK1/2 in response to the β_2_-agonist isoprenaline. Interestingly, however, AKAP12-mediated recycling of the β_2_-adrenoceptor was unaffected upon AKAP5 overexpression [31]. We have recently published a hypothetical model how AKAP5, AKAP12, and Ezrin can work in harmony to regulate that β_2_-adrenoceptor expression at the membrane [80]. However, mechanisms involved in AKAP dimerization, and how such dimer formation is triggered by molecular cues still remain obscure. At last, studies referring to the roles of some AKAPs (e.g., AKAP8) in diseases are very limited. Nevertheless, further studies are necessary to help us gain more knowledge about the role of AKAPs in cell cycle regulation, therefore providing new insights to perhaps develop AKAP-related therapies to treat diseases caused by abnormal cell cycle regulation.

## References

[1] Akakura S, Gelman IH (2012) Pivotal role of AKAP12 in the regulation of cellular adhesion dynamics: control of cytoskeletal architecture, cell migration, and mitogenic signaling. J Signal Transduct 529179. doi:10.1155/2012/529179 10.1155/2012/529179 PMC3395252 22811901

[2] Akakura S, Nochajski P, Gao L, Sotomayor P, Matsui S, Gelman IH (2010). Rb-dependent cellular senescence, multinucleation and susceptibility to oncogenic transformation through PKC scaffolding by SSeCKS/AKAP12. Cell Cycle.

[3] Akakura S, Huang C, Nelson PJ, Foster B, Gelman IH (2008). Loss of the SeCKS/Gravin/AKAP12 gene results in prostatic hyperplasia. Cancer Res.

[4] Alberts B, Johnson A, Lewis J, Raff M, Roberts K, Walter P (2002) Molecular biology of the cell. Garland Science, New York

[5] Alfthan K, Heiska L, Gronholm M, Renkema GH, Carpen O (2004). Cyclic AMP-dependent protein kinase phosphorylates Merlin at serine 518 independently of p21-activated kinase and promotes Merlin-Ezrin heterodimerization. J Biol Chem.

[6] Anjum R, Blenis J (2008). The RSK family of kinases: emerging roles in cellular signalling. Nat Rev Mol Cell Biol.

[7] Arsenijevic T, Degraef C, Dumont JE, Roger PP, Pirson I (2006). G1/S cyclins interact with regulatory subunit of PKA via A-kinase anchoring protein, AKAP95. Cell Cycle.

[8] Arsenijevic T, Degraef C, Dumont JE, Roger PP, Pirson I (2004). A novel partner for D-type cyclins: protein kinase A-anchoring protein AKAP95. Biochem J.

[9] Bement WM, Capco DG (1991). Analysis of inducible contractile rings suggests a role for protein kinase C in embryonic cytokinesis and wound healing. Cell Motil Cytoskeleton.

[10] Bertoli C, Skotheim JM, de Bruin RA (2013). Control of cell cycle transcription during G1 and S phases. Nat Rev Mol Cell Biol.

[11] Billington CK, Ojo OO, Penn RB, Ito S (2013). cAMP regulation of airway smooth muscle function. Pulm Pharmacol Ther.

[12] Burnworth B, Pippin J, Karna P (2012). SSeCKS sequesters cyclin D1 in glomerular parietal epithelial cells and influences proliferative injury in the glomerulus. Lab Invest.

[13] Canton DA, Scott JD (2013). Anchoring proteins encounter mitotic kinases. Cell Cycle.

[14] Canton DA, Keene CD, Swinney K (2012). Gravin is a transitory effector of polo-like kinase 1 during cell division. Mol Cell.

[15] Caretta A, Mucignat-Caretta C (2011). Protein kinase A in cancer. Cancers (Basel).

[16] Chambard JC, Lefloch R, Pouyssegur J, Lenormand P (2007). ERK implication in cell cycle regulation. Biochim Biophys Acta.

[17] Chen H, Mei L, Zhou L (2011). Moesin-Ezrin-Radixin-like protein (Merlin) mediates protein interacting with the carboxyl terminus-1 (PICT-1)-induced growth inhibition of glioblastoma cells in the nucleus. Int J Biochem Cell Biol.

[18] Chen QY, Xu W, Jiao DM, Wu LJ, Song J, Yan J, Shi JG (2013). Silence of Ezrin modifies migration and actin cytoskeleton rearrangements and enhances chemosensitivity of lung cancer cells in vitro. Mol Cell Biochem.

[19] Choi MC, Lee YU, Kim SH (2008). A-kinase anchoring protein 12 regulates the completion of cytokinesis. Biochem Biophys Res Commun.

[20] Choi MC, Jong HS, Kim TY (2004). AKAP12/Gravin is inactivated by epigenetic mechanism in human gastric carcinoma and shows growth suppressor activity. Oncogene.

[21] Collas P, Le Guellec K, Tasken K (1999). The A-kinase-anchoring protein AKAP95 is a multivalent protein with a key role in chromatin condensation at mitosis. J Cell Biol.

[22] Daaka Y, Luttrell LM, Lefkowitz RJ (1997). Switching of the coupling of the beta2-adrenergic receptor to different G proteins by protein kinase A. Nature.

[23] Deak VA, Klussmann E (2015) Pharmacological interference with protein-protein interactions of A-kinase anchoring proteins as a strategy for the treatment of disease. Curr Drug Targets 10.2174/1389450116666150416114247 25882214

[24] Dick FA, Rubin SM (2013). Molecular mechanisms underlying RB protein function. Nat Rev Mol Cell Biol.

[25] Dransfield DT, Bradford AJ, Smith J, Martin M, Roy C, Mangeat PH, Goldenring JR (1997). Ezrin is a cyclic AMP-dependent protein kinase anchoring protein. EMBO J.

[26] Dumaz N, Marais R (2005). Integrating signals between cAMP and the RAS/RAF/MEK/ERK signalling pathways. Based on the anniversary prize of the Gesellschaft fur Biochemie und Molekularbiologie Lecture delivered on 5 July 2003 at the Special FEBS Meeting in Brussels. FEBS J.

[27] Ebisuya M, Kondoh K, Nishida E (2005). The duration, magnitude and compartmentalization of ERK MAP kinase activity: mechanisms for providing signaling specificity. J Cell Sci.

[28] Eide T, Tasken KA, Carlson C, Williams G, Jahnsen T, Tasken K, Collas P (2003). Protein kinase A-anchoring protein AKAP95 interacts with MCM2, a regulator of DNA replication. J Biol Chem.

[29] Esseltine JL, Scott JD (2013). AKAP signaling complexes: pointing towards the next generation of therapeutic targets?. Trends Pharmacol Sci.

[30] Fehon RG, McClatchey AI, Bretscher A (2010). Organizing the cell cortex: the role of ERM proteins. Nat Rev Mol Cell Biol.

[31] Gao S, Wang HY, Malbon CC (2011) AKAP12 and AKAP5 form higher-order hetero-oligomers. J Mol Signal 8-2187-6-8. doi:10.1186/1750-2187-6-8 10.1186/1750-2187-6-8 PMC3170326 21831305

[32] Gao X, Chaturvedi D, Patel TB (2012). Localization and retention of p90 ribosomal S6 kinase 1 in the nucleus: implications for its function. Mol Biol Cell.

[33] Gelman IH (2012). Suppression of tumor and metastasis progression through the scaffolding functions of SSeCKS/Gravin/AKAP12. Cancer Metastasis Rev.

[34] Gelman IH (2010). Emerging roles for SSeCKS/Gravin/AKAP12 in the control of cell proliferation, cancer malignancy, and barriergenesis. Genes Cancer.

[35] Gordon T, Grove B, Loftus JC, O’Toole T, McMillan R, Lindstrom J, Ginsberg MH (1992). Molecular cloning and preliminary characterization of a novel cytoplasmic antigen recognized by myasthenia gravis sera. J Clin Invest.

[36] Grieco D, Porcellini A, Avvedimento EV, Gottesman ME (1996). Requirement for cAMP-PKA pathway activation by M phase-promoting factor in the transition from mitosis to interphase. Science.

[37] Grieco D, Avvedimento EV, Gottesman ME (1994). A role for cAMP-dependent protein kinase in early embryonic divisions. Proc Natl Acad Sci U S A.

[38] Gronholm M, Muranen T, Toby GG, Utermark T, Hanemann CO, Golemis EA, Carpen O (2006). A functional association between Merlin and HEI10, a cell cycle regulator. Oncogene.

[39] Gronholm M, Vossebein L, Carlson CR (2003). Merlin links to the cAMP neuronal signaling pathway by anchoring the RIbeta subunit of protein kinase A. J Biol Chem.

[40] Guo LW, Gao L, Rothschild J, Su B, Gelman IH (2011). Control of protein kinase C activity, phorbol ester-induced cytoskeletal remodeling, and cell survival signals by the scaffolding protein. SSeCKS/GRAVIN/AKAP12. J Biol Chem.

[41] Hebert AM, DuBoff B, Casaletto JB, Gladden AB, McClatchey AI (2012). Merlin/ERM proteins establish cortical asymmetry and centrosome position. Genes Dev.

[42] Hendzel MJ, Wei Y, Mancini MA (1997). Mitosis-specific phosphorylation of histone H3 initiates primarily within pericentromeric heterochromatin during G2 and spreads in an ordered fashion coincident with mitotic chromosome condensation. Chromosoma.

[43] Herberg FW, Maleszka A, Eide T, Vossebein L, Tasken K (2000). Analysis of A-kinase anchoring protein (AKAP) interaction with protein kinase A (PKA) regulatory subunits: PKA isoform specificity in AKAP binding. J Mol Biol.

[44] Hewer RC, Sala-Newby GB, Wu YJ, Newby AC, Bond M (2011). PKA and Epac synergistically inhibit smooth muscle cell proliferation. J Mol Cell Cardiol.

[45] Iida S, Hirota T, Morisaki T (2004). Tumor suppressor WARTS ensures genomic integrity by regulating both mitotic progression and G1 tetraploidy checkpoint function. Oncogene.

[46] Indolfi C, Stabile E, Coppola C (2001). Membrane-bound protein kinase A inhibits smooth muscle cell proliferation in vitro and in vivo by amplifying cAMP-protein kinase A signals. Circ Res.

[47] Jarnaess E, Ruppelt A, Stokka AJ, Lygren B, Scott JD, Tasken K (2008). Dual specificity A-kinase anchoring proteins (AKAPs) contain an additional binding region that enhances targeting of protein kinase A type I. J Biol Chem.

[48] Jin Z, Hamilton JP, Yang J (2008). Hypermethylation of the AKAP12 promoter is a biomarker of Barrett’s-associated esophageal neoplastic progression. Cancer Epidemiol Biomarkers Prev.

[49] Kang YK, Hong SW, Lee H, Kim WH (2010). Prognostic implications of Ezrin expression in human hepatocellular carcinoma. Mol Carcinog.

[50] Keryer G, Yassenko M, Labbe JC, Castro A, Lohmann SM, Evain-Brion D, Tasken K (1998). Mitosis-specific phosphorylation and subcellular redistribution of the RIIalpha regulatory subunit of cAMP-dependent protein kinase. J Biol Chem.

[51] Kishore R, Qin G, Luedemann C (2005). The cytoskeletal protein Ezrin regulates EC proliferation and angiogenesis via TNF-alpha-induced transcriptional repression of cyclin A. J Clin Invest.

[52] Kishore R, Spyridopoulos I, Luedemann C, Losordo DW (2002). Functionally novel tumor necrosis factor-alpha-modulated CHR-binding protein mediates cyclin A transcriptional repression in vascular endothelial cells. Circ Res.

[53] Kong J, Li Y, Liu S et al. (2013) High expression of Ezrin predicts poor prognosis in uterine cervical cancer BMC. Cancer 520-2407-13-520. doi:10.1186/1471-2407-13-520 10.1186/1471-2407-13-520 PMC4228363 24182314

[54] Landsverk HB, Carlson CR, Steen RL, Vossebein L, Herberg FW, Tasken K, Collas P (2001). Regulation of anchoring of the RIIalpha regulatory subunit of PKA to AKAP95 by threonine phosphorylation of RIIalpha: implications for chromosome dynamics at mitosis. J Cell Sci.

[55] Laulajainen M, Muranen T, Carpen O, Gronholm M (2008). Protein kinase A-mediated phosphorylation of the NF2 tumor suppressor protein Merlin at serine 10 affects the actin cytoskeleton. Oncogene.

[56] Lei M, Tye BK (2001). Initiating DNA synthesis: from recruiting to activating the MCM complex. J Cell Sci.

[57] Leiphrakpam PD, Rajput A, Mathiesen M (2014). Ezrin expression and cell survival regulation in colorectal cancer. Cell Signal.

[58] Li Y, Kao GD, Garcia BA (2006). A novel histone deacetylase pathway regulates mitosis by modulating Aurora B kinase activity. Genes Dev.

[59] Lin X, Gelman IH (2002). Calmodulin and cyclin D anchoring sites on the Src-suppressed C kinase substrate, SSeCKS. Biochem Biophys Res Commun.

[60] Lin X, Nelson P, Gelman IH (2000). SSeCKS, a major protein kinase C substrate with tumor suppressor activity, regulates G(1)-->S progression by controlling the expression and cellular compartmentalization of cyclin D. Mol Cell Biol.

[61] Lin X, Gelman IH (1997). Reexpression of the major protein kinase C substrate, SSeCKS, suppresses v-src-induced morphological transformation and tumorigenesis. Cancer Res.

[62] Lin X, Nelson PJ, Frankfort B, Tombler E, Johnson R, Gelman IH (1995). Isolation and characterization of a novel mitogenic regulatory gene, 322, which is transcriptionally suppressed in cells transformed by src and ras. Mol Cell Biol.

[63] Liu H, Huang X, Wang H, Shen A, Cheng C (2009). Dexamethasone inhibits proliferation and stimulates SSeCKS expression in C6 rat glioma cell line. Brain Res.

[64] Mak H, Naba A, Varma S et al. (2012) Ezrin phosphorylation on tyrosine 477 regulates invasion and metastasis of breast cancer cells BMC. Cancer 82-2407-12-82. doi:10.1186/1471-2407-12-82 10.1186/1471-2407-12-82 PMC3372425 22397367

[65] Makitie T, Carpen O, Vaheri A, Kivela T (2001). Ezrin as a prognostic indicator and its relationship to tumor characteristics in uveal malignant melanoma. Invest Ophthalmol Vis Sci.

[66] Mao J, Yuan XR, Xu SS, Jiang XC, Zhao XT (2013). Expression and functional significance of Ezrin in human brain astrocytoma. Cell Biochem Biophys.

[67] McCormick K, Baillie GS (2014). Compartmentalisation of second messenger signalling pathways. Curr Opin Genet Dev.

[68] McCubrey JA, Steelman LS, Chappell WH (2007). Roles of the Raf/MEK/ERK pathway in cell growth, malignant transformation and drug resistance. Biochim Biophys Acta.

[69] Meng Y, Lu Z, Yu S, Zhang Q, Ma Y, Chen J (2010) Ezrin promotes invasion and metastasis of pancreatic cancer cells. J. Transl. Med. 61-5876-8-61. doi:10.1186/1479-5876-8-61 10.1186/1479-5876-8-61 PMC2916894 20569470

[70] Mori Y, Cai K, Cheng Y (2006). A genome-wide search identifies epigenetic silencing of somatostatin, Tachykinin-1, and 5 other genes in colon cancer. Gastroenterology.

[71] Morrison H, Sherman LS, Legg J (2001). The NF2 tumor suppressor gene product, merlin, mediates contact inhibition of growth through interactions with CD44. Genes Dev.

[72] Muranen T, Gronholm M, Renkema GH, Carpen O (2005). Cell cycle-dependent nucleocytoplasmic shuttling of the neurofibromatosis 2 tumour suppressor Merlin. Oncogene.

[73] Nauert JB, Klauck TM, Langeberg LK, Scott JD (1997). Gravin, an autoantigen recognized by serum from myasthenia gravis patients, is a kinase scaffold protein. Curr Biol.

[74] Nigg EA (1995). Cyclin-dependent protein kinases: key regulators of the eukaryotic cell cycle. Bioessays.

[75] Ohtani K, Sakamoto H, Rutherford T (2002). Ezrin, a membrane-cytoskeletal linking protein, is highly expressed in atypical endometrial hyperplasia and uterine endometrioid adenocarcinoma. Cancer Lett.

[76] Page K, Li J, Hershenson MB (1999). Platelet-derived growth factor stimulation of mitogen-activated protein kinases and cyclin D1 promoter activity in cultured airway smooth-muscle cells. Role of Ras Am J Respir Cell Mol Biol.

[77] Pellagatti A, Cazzola M, Giagounidis A (2010). Deregulated gene expression pathways in myelodysplastic syndrome hematopoietic stem cells. Leukemia.

[78] Petrilli AM, Fernández-Valle C (2015). Role of Merlin/NF2 inactivation in tumor biology. Oncogene.

[79] Poppinga WJ, Heijink IH, Holtzer LJ (2015). A-kinase-anchoring proteins coordinate inflammatory responses to cigarette smoke in airway smooth muscle. Am J Physiol Lung Cell Mol Physiol.

[80] Poppinga WJ, Munoz-Llancao P, Gonzalez-Billault C, Schmidt M (2014). A-kinase anchoring proteins: cyclic AMP compartmentalization in neurodegenerative and obstructive pulmonary diseases. Br J Pharmacol.

[81] Raman M, Chen W, Cobb MH (2007). Differential regulation and properties of MAPKs. Oncogene.

[82] Ravenhall C, Guida E, Harris T, Koutsoubos V, Stewart A (2000). The importance of ERK activity in the regulation of cyclin D1 levels and DNA synthesis in human cultured airway smooth muscle. Br J Pharmacol.

[83] Ren L, Hong SH, Chen QR (2012). Dysregulation of Ezrin phosphorylation prevents metastasis and alters cellular metabolism in osteosarcoma. Cancer Res.

[84] Rouleau GA, Merel P, Lutchman M (1993). Alteration in a new gene encoding a putative membrane-organizing protein causes neuro-fibromatosis type 2. Nature.

[85] Rozenblatt-Rosen O, Mosonego-Ornan E, Sadot E, Madar-Shapiro L, Sheinin Y, Ginsberg D, Yayon A (2002). Induction of chondrocyte growth arrest by FGF: transcriptional and cytoskeletal alterations. J Cell Sci.

[86] Saito S, Yamamoto H, Mukaisho K (2013). Mechanisms underlying cancer progression caused by Ezrin overexpression in tongue squamous cell carcinoma. PLoS One.

[87] Saurin AT, Durgan J, Cameron AJ, Faisal A, Marber MS, Parker PJ (2008). The regulated assembly of a PKCepsilon complex controls the completion of cytokinesis. Nat Cell Biol.

[88] Schmidt M, Dekker FJ, Maarsingh H (2013). Exchange protein directly activated by cAMP (epac): a multidomain cAMP mediator in the regulation of diverse biological functions. Pharmacol Rev.

[89] Schmitt JM, Stork PJ (2000). Beta 2-adrenergic receptor activates extracellular signal-regulated kinases (ERKs) via the small G protein rap1 and the serine/threonine kinase B-Raf. J Biol Chem.

[90] Sheppard CL, Lee LC, Hill EV (2014). Mitotic activation of the DISC1-inducible cyclic AMP phosphodiesterase-4D9 (PDE4D9), through multi-site phosphorylation, influences cell cycle progression. Cell Signal.

[91] Skroblin P, Grossmann S, Schafer G, Rosenthal W, Klussmann E (2010). Mechanisms of protein kinase A anchoring. Int Rev Cell Mol Biol.

[92] Smith FD, Langeberg LK, Cellurale C, Pawson T, Morrison DK, Davis RJ, Scott JD (2010). AKAP-Lbc enhances cyclic AMP control of the ERK1/2 cascade. Nat Cell Biol.

[93] Sperka T, Wang J, Rudolph KL (2012). DNA damage checkpoints in stem cells, ageing and cancer. Nat Rev Mol Cell Biol.

[94] Steen RL, Cubizolles F, Le Guellec K, Collas P (2000). A kinase-anchoring protein (AKAP)95 recruits human chromosome-associated protein (hCAP)-D2/Eg7 for chromosome condensation in mitotic extract. J Cell Biol.

[95] Su B, Gao L, Meng F, Guo LW, Rothschild J, Gelman IH (2013). Adhesion-mediated cytoskeletal remodeling is controlled by the direct scaffolding of Src from FAK complexes to lipid rafts by SSeCKS/AKAP12. Oncogene.

[96] Takahashi A, Ohtani N, Hara E (2007). Irreversibility of cellular senescence: dual roles of p16INK4a/Rb-pathway in cell cycle control. Cell Div.

[97] Tao T, Ji Y, Cheng C (2009). Tumor necrosis factor-alpha inhibits Schwann cell proliferation by up-regulating Src-suppressed protein kinase C substrate expression. J Neurochem.

[98] Therrien M, Michaud NR, Rubin GM, Morrison DK (1996). KSR modulates signal propagation within the MAPK cascade. Genes Dev.

[99] Toby GG, Gherraby W, Coleman TR, Golemis EA (2003). A novel RING finger protein, human enhancer of invasion 10, alters mitotic progression through regulation of cyclin B levels. Mol Cell Biol.

[100] Toyoshima-Morimoto F, Taniguchi E, Nishida E (2002). Plk1 promotes nuclear translocation of human Cdc25C during prophase. EMBO Rep.

[101] Toyoshima-Morimoto F, Taniguchi E, Shinya N, Iwamatsu A, Nishida E (2001). Polo-like kinase 1 phosphorylates cyclin B1 and targets it to the nucleus during prophase. Nature.

[102] Trofatter JA, MacCollin MM, Rutter JL (1993). A novel Moesin-, Ezrin-, Radixin-like gene is a candidate for the neurofibromatosis 2 tumor suppressor. Cell.

[103] Troger J, Moutty MC, Skroblin P, Klussmann E (2012). A-kinase anchoring proteins as potential drug targets. Br J Pharmacol.

[104] Vandame P, Spriet C, Trinel D (2014). The spatio-temporal dynamics of PKA activity profile during mitosis and its correlation to chromosome segregation. Cell Cycle.

[105] Villanueva J, Yung Y, Walker JL, Assoian RK (2007). ERK activity and G1 phase progression: identifying dispensable versus essential activities and primary versus secondary targets. Mol Biol Cell.

[106] Wan PT, Garnett MJ, Roe SM (2004). Mechanism of activation of the RAF-ERK signaling pathway by oncogenic mutations of B-RAF. Cell.

[107] Wikman H, Kettunen E, Seppanen JK, Karjalainen A, Hollmen J, Anttila S, Knuutila S (2002). Identification of differentially expressed genes in pulmonary adenocarcinoma by using cDNA array. Oncogene.

[108] Wilkins BJ, Rall NA, Ostwal Y (2014). A cascade of histone modifications induces chromatin condensation in mitosis. Science.

[109] Wong W, Scott JD (2004). AKAP signalling complexes: focal points in space and time. Nat Rev Mol Cell Biol.

[110] Yamazaki D, Kurisu S, Takenawa T (2005). Regulation of cancer cell motility through actin reorganization. Cancer Sci.

[111] Yang X, Yu K, Hao Y, Li DM, Stewart R, Insogna KL, Xu T (2004). LATS1 tumour suppressor affects cytokinesis by inhibiting LIMK1. Nat Cell Biol.

[112] Yasutis KM, Kozminski KG (2013). Cell cycle checkpoint regulators reach a zillion. Cell Cycle.

[113] Zakhary DR, Fink MA, Ruehr ML, Bond M (2000). Selectivity and regulation of A-kinase anchoring proteins in the heart. The role of autophosphorylation of the type II regulatory subunit of cAMP-dependent protein kinase. J Biol Chem.

[114] Zhang Q, Carr DW, Lerea KM, Scott JD, Newman SA (1996). Nuclear localization of type II cAMP-dependent protein kinase during limb cartilage differentiation is associated with a novel developmentally regulated A-kinase anchoring protein. Dev Biol.

[115] Zhivotovsky B, Orrenius S (2010). Cell cycle and cell death in disease: past, present and future. J Intern Med.

[116] Zhou J, Feng Y, Tao K (2014). The expression and phosphorylation of Ezrin and Merlin in human pancreatic cancer. Int J Oncol.

[117] Zitouni S, Nabais C, Jana SC, Guerrero A, Bettencourt-Dias M (2014). Polo-like kinases: structural variations lead to multiple functions. Nat Rev Mol Cell Biol.

